# Association between sexual violence and unintended pregnancy among married women in Zambia

**DOI:** 10.1186/s12889-022-13881-8

**Published:** 2022-08-05

**Authors:** Mwewa E. Kasonde, Bwalya Bupe Bwalya, Elizabeth T. Nyirenda, Chabila Christopher Mapoma, Milika Sikaluzwe, Kafiswe Chimpinde, Gloria I. Songolo

**Affiliations:** 1 Department of Population Studies, School of Humanities and Social Sciences, University of Zambia, Lusaka, Zambia; 2 Directorate of Research and Postgraduate Studies, Mulungushi University, Kabwe, Zambia

**Keywords:** Unintended pregnancy, Sexual violence, Married women, Contraceptive use, Zambia

## Abstract

**Background:**

One of the outcomes of sexual violence is unintended pregnancy. In Zambia, 15% of married women age 15—49 years had experienced sexual violence from their husband or partner. The prevalence of unintended pregnancies among women age 15—49 years has risen from 33% in 1992 to 38% in 2018. The link between sexual violence and unintended pregnancy in Zambia was investigated in this study.

**Methods:**

This study used the women's dataset from the 2018 Zambia Demographic and Health Survey, a cross-sectional survey. The study looked at a weighted sample size of 4,465 women age 15 – 49 years. Unintended pregnancy was measured by combining response categories of mistimed and unwanted pregnancy. Multivariate binary logistic regression was performed to establish the net effects of sexual violence and each explanatory variable on unintended pregnancy.

**Results:**

The findings suggest that sexual violence does have a role in unintended pregnancies (AOR 1.74; CI 1.38—2.19). Ever use of contraception is also a significant predictor of unintended pregnancy (AOR 1.48; CI 1.16—1.88), even when other characteristics are taken into account. Results have shown that a woman who had ever used contraception and had experienced sexual violence was more likely to have an unintended pregnancy.

**Conclusion:**

Spousal sexual violence is highly associated with unintended pregnancies in Zambia. Addressing intimate partner sexual violence is among the ways to prevent unintended pregnancies. It is also important to sensitize women on reporting acts of sexual violence to relevant authorities as this will not only prevent reoccurrence of sexual violence but also reduce unintended pregnancies and associated long-term effects.

## Background

Violence against women is a human rights violation as well as a global public health issue [1]. Particularly, sexual violence perpetuated by intimate partners continues to occur throughout the world especially among women. Sexual violence is defined as “any sexual act, attempt to obtain a sexual act, or other acts directed against a person’s sexuality using coercion, by any person regardless of their relationship to the victim, in any setting” [2]. It includes rape, attempted rape, unwanted sexual touching and other non-contact forms” [3]. In this study, we focus on sexual violence perpetrated by a husband/partner.

Globally, 1 in 3 women in 2018 were estimated to have experienced physical and/or sexual violence by an intimate partner or sexual violence by any perpetrator in their lifetime [2]. The 2018 prevalence estimates of lifetime intimate partner violence (IPV) ranges from 20% in the Western Pacific to 33% in the World Health Organisation (WHO) African Region and WHO South-East Asia Region, respectively [3]. Sexual violence against women and girls not only violates their rights, but limits their ability to participate in society as well and essentially reduces their health and well-being [4]. In addition, spousal sexual violence affects a woman's physical, mental, sexual, and reproductive health, as well as her ability to make decisions [5]. Likely effects include increased risk of sexually transmitted infections within married couples through forced unforced unprotected sexual intercourse, urinary tract infections and sexual dysfunction [2].

Unintended pregnancies have also been linked to sexual violence [6, 7]. In this study, unintended pregnancy was defined as “a pregnancy that is either unwanted, where a pregnancy occurred when no children or no more children were desired, or the pregnancy is mistimed, where the pregnancy occurred earlier than desired” [8]. There were 121 million unintended pregnancies worldwide annually, between 2015–2019 (uncertainty intervals (UIs) 112.8–131.5), corresponding to a global rate of 64 unintended pregnancies (UI 60–70) per 1,000 women in the age group 15–49 [9]. Unintended pregnancy rates among women age 15–49 years vary by area, ranging from 35 pregnancies (UI 33–39) in Europe and Northern America to 91 pregnancies (UI 86–96) in Sub-Saharan Africa [9].

IPV history and experience of spousal violence (physical or sexual violence) have both been found to be associated with unwanted pregnancies [4, 10, 11]. This is because IPV fosters an environment that impacts a woman's autonomy, participation in decision-making related to her own health care, availability and use of contraceptives, and bargaining for safe sex, leading to forced unprotected sex and unexpected pregnancy [12, 13].

Unwanted pregnancy has also been attributable to a desire to have more children, a lack of contraceptive knowledge, spouse disapproval of contraception, difficulties in obtaining contraceptives, and contraceptive technique failure [14]. Unwanted pregnancies and mistimed or unwanted births may affect women’s health negatively; this negative effect extends to both the well-being of children and family alike. Mistimed or unplanned pregnancies and births have a variety of implications, ranging from socioeconomic to physiological. The lack of access to safe abortions exposes women to unsafe abortions or unintended births due to barriers and challenges such as restrictive abortion laws, ignorance of the existence of abortion law and what is permitted, and cultural and societal stigma associated with abortion [15]. Delayed initiation of antenatal care [16]; maternal depression due to unintended pregnancy is not uncommon too [17]. In some situations, young mothers drop out of school as they are required to take care of their own children, thereby increasing the burden of care on families [18].

Spousal sexual violence and unintended pregnancies are also prevalent in the country of this study, Zambia. In 2018, 15% of currently married women age 15–49 reported ever-experiencing sexual violence by husband or partner [19]. The 2018 Zambia Demographic and Health Survey (ZDHS) also shows an increase in unplanned pregnancies from 33% in 1992 to 38% in 2018. This scenario supports already provided evidence where there exists linkages between sexual violence and unintended pregnancies [4, 13, 20].

The literature reviewed shows that there is a paltry of evidence on studies focusing on understanding the association between spousal sexual violence and unintended pregnancies in Zambia. Some studies that may have been conducted on this subject were based on teenage and adolescent fertility, contraceptive use, HIV and unintended pregnancies [21–23]. However, there is a dearth of evidence that IPV specifically sexual violence affects women’s fertility and evidence further shows that very few studies have explored the relationship between sexual violence and women’s ability to control their fertility especially in developing countries like Zambia [10]. Thus, this study explored the association between sexual violence by husband /partner and unintended pregnancies in Zambia using data from a nationally representative sample based on the 2018 DHS.

**Fig. 1:**
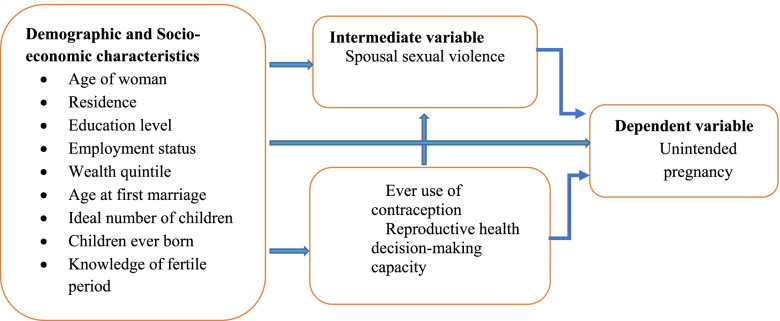
Conceptual framework of unintended pregnancy and sexual violence by husband or partner

The developed conceptual framework in Fig. 1 attempts to demonstrate the association between sexual violence by husband or partner and unintended pregnancy in Zambia. The main predictor variable in this study was sexual violence, influenced as well by socio-economic and demographic factors such age, education level, wealth status among many others. Women who have ever experienced sexual violence by husband or partner are more likely to report unintended pregnancies. Furthermore, demographic and socio-economic characteristics influence women’s likelihood of experiencing sexual violence from husband or partner, ever use of contraception and reproductive health decision-making capacity and these may also contribute directly or indirectly to unintended pregnancies.

## Methods

### Data source

This is an exploratory cross-sectional study aimed at establishing association between sexual violence and unintended pregnancy based on the analysis of data from the 2018 ZDHS. The ZDHS was conducted in selected urban and rural clusters in all the ten provinces in Zambia from 17 July 2018 to 24 January 2019. The ZDHS was a nationally representative survey with a sample designed to produce estimates on a range of basic demographic and health indicators at the national and provincial levels, as well as by residence (rural and urban areas). The sampling method of the ZDHS has been reported in detail elsewhere [ZDHS, 2018]. The current study analysed data gathered from the women’s record questionnaire, where such aspects as women’s background characteristics; family planning; Antenatal, delivery, and postnatal care; Maternal and child health nutrition; marriage and sexual activity; reproduction, fertility preferences; domestic violence; women’s work and husband’s background characteristics are collected. All methods in our study were carried out in accordance with relevant guidelines and regulations of using DHS data and BMC Public Health Journal guidelines in preparing manuscripts.

### Sample design and sampling procedure

The 2018 ZDHS used a stratified two-stage sample design. Sample clusters consisting of Enumeration Area (EAs) were selected with a probability proportional to their size within each sampling stratum and 545 clusters (198 in urban areas and 347 in rural areas) were selected at the first stage. The second stage involved systematic sampling of households in all of the selected clusters. An average of 133 households per cluster were found during household listing and from which 25 households were selected through an equal probability systematic selection process, to obtain a total sample size of 13,625 households. All women age 15–49 and men age 15–59 who were either permanent residents of the selected households or visitors who stayed in the households the night before the survey were eligible for interviews. During the 2018 ZDHS, written informed consent was obtained from all the selected eligible persons for interviews. This means all participants included in the current study had provided written informed consent. Through written request, the authors obtained approval from the DHS Program to use the 2018 ZDHS woman’s recode.

### Target population and sample size

The population for this study consisted of women in the age group 15–49 selected and interviewed on the domestic violence module and gave birth in the last 5 years before the survey. Implementation of the domestic violence module followed the World Health Organization’s guidelines on the ethical collection of information on domestic violence, where only one eligible woman per household was randomly selected for interviewing after obtaining informed consent. Subsequently, out of the 13,683 interviewed on the other topics, 9,503 women were successfully interviewed on the domestic violence module. However, the final weighted sample size for this study meeting the inclusion criteria of being married, having responded to the questions on domestic violence and having a birth in the five years prior to the survey was 4,465.

### Study variables

#### Outcome Variable(s)

The outcome variable is unintended pregnancy (for the most recent pregnancy in the five years prior to the survey). It was created from a question where women who had given birth in the five years prior to the survey for the recent pregnancy were asked if they wanted the pregnancy when they became pregnant. The question has three response categories: wanted then, wanted later, no more. The outcome variable in this study “unintended pregnancy” was created with two categories: where “0” or “Intended” was for all women who said the most recent pregnancy was wanted; and “1”or “Unintended” combined women that wanted to be pregnant but later (mistimed) and those women who were pregnant but did not want any more pregnancy (unwanted).

### Predictor variables

#### Main predictor variable

The main predictor variable was sexual violence. It was measured by asking ever-married women a series of questions including the following: Did your (last) (husband/partner) ever do any of the following things to you: 1) physically force you to have sexual intercourse with him when you did not want to? 2) Physically force you to perform any other sexual acts you did not want to? 3) Force you with threats or in any other way to perform sexual acts you did not want to? [19]. Sexual violence was coded “0” or “No” (no experience of sexual violence by husband/ partner if the response was “No” to all of these questions), and “1” or “Yes” (experienced sexual violence by husband/ partner if Yes to one or more of these questions above).

#### Other predictor variables

Other predictor variables included socio-economic and demographic characteristics of women such as age at last birthday in years (15–19, 20–24, 25–29, 30–34, 35–39, 40–44 and 45–49), number of living children (0, 1–2, 3–4, 5 +), education level (no education, primary, secondary and higher), wealth status (poor, middle, rich), employment status (not working and working), and residence (rural and urban).

#### Mediator variables

For purposes of this study, two variables were identified as possible mediators, namely ever use of any contraception among married couples before getting pregnant and women’s reproductive health decision making capacity (RHDMC) among respondents. Ever used any contraceptive was coded: those who were currently using and those who were not currently using but had used before were classified as “Yes” ever used whilst those who had never used any method were classified as “No”.

RHDMC was derived from two variables namely: 1) decision-making on sexual intercourse, that is women were asked whether they could say no to their husband if they do not want to have sexual intercourse: 2) decision-making on condom use, that is women were asked whether they could ask their husband to use a condom during sexual intercourse [24]. Each one of these questions had three response categories namely, yes, no and do not know. Therefore, the variable “reproductive health decision-making capacity” was generated as a two outcome variable, with women who said “no and don’t know” to both questions recoded as “No” implying not capable of making reproductive health decision whilst those who said “Yes'' to any or both questions were recoded as “Yes” and labelled as capable of making reproductive health decisions.

### Statistical analysis

A calculated special weight for domestic violence for the 2018 ZDHS which accounts for the selection of one woman per household and for module non-response was applied to ensure that the sample was nationally representative using the svyset command to account for complex survey design. These weights were calculated by multiplying the household sampling weight from which the woman was sampled by the inverse of the woman's individual response rate by stratum, and then normalising the results to obtain the final standard weights used in this analysis by multiplying the sampling weight by the estimated sampling fraction obtained from the survey for the household weight and the individual woman's weight.

Data analysis was conducted using Stata version 14 where univariate, bivariate and multivariate binary logistic regression were performed. Univariate analysis produced distribution of women by different demographic and socio-economic factors. Chi-square test was performed to examine if there was association between the outcome variable – unintended pregnancy and the main predictor variable – sexual violence and other independent variables.

Multivariate binary logistic regression was performed to determine adjusted effects of sexual violence on unintended pregnancy adjusted for demographic and socio-economic factors. For our study, we performed four models (i) Unadjusted Odds Ratio (UOR) for women’s experience of sexual violence on unintended pregnancy; (ii) Adjusted Odds Ratio (AORs) of women’s ever use of contraceptive method and reproductive health decision-making capacity on unintended pregnancy; (iii) AORs for women’s experience of sexual violence, ever use of contraceptive method and reproductive health decision-making capacity on unintended pregnancy; and (iv) AORs for women’s experience of sexual violence, ever use of contraceptive method, reproductive health decision-making capacity and demographic and socio-economic characteristics on unintended pregnancy. Both the UORs and AORs were considered significant at *p* < 0.001, *p* < 0.01 and *p* < 0.05.

Further, in order to for us to evaluate the adjusted associations between the predictor variable (Sexual Violence), mediating factors (Ever use of contraception and RHDMC), and the outcome variable (unintended pregnancies), we constructed three model path ways based on the Generalised Structural Equation Modelling (GSEM) Stata ‘gsem’ command. All variables, predictor, mediating and outcome variables were coded as binary variables and as such, all path models (a, b, c and d) were modelled as logistic models [25]. As such, mediation existed in our modelling when the predicted variable was simultaneously regressed onto the predicting variable and the mediator.

## Results

### Background characteristics of women

Of the 13, 683 women surveyed in the 2018 ZDHS, 4,465 were married, responded to the questions on domestic violence and had a birth in the five years prior to the survey. Of these, 25% were 25–29 years, 63% reside in rural areas, 51% attained primary level education and 45% lived in households classified as poor (Table 1). The results also show that 50% were employed, 54% were married for the first time when they were age 18 or older and 37% had 1 to 2 children with more than half (53%) having an ideal number of five and/or more children. The results further show that 50% of the women had reproductive health decision-making capacity and the other 50% did not. In addition, 8 in 10 women reported ever used any method of contraceptive and did not know the fertile period, 85% of the women had not experienced spousal sexual violence (Table 1). The results from this analysis shows that 36% of the pregnancies were unintended.

**Table 1:** Socio-economic and demographic characteristics of women

Characteristics	ZDHS 2018	
	%	n
**Mean age (in year**s)		29.3
**Age group**		
15–19	5.3	238
20–24	23.1	1,031
25–29	24.6	1,098
30–34	20.5	915
35–39	16.1	720
40–44	7.7	345
45–49	2.7	118
**Type of residence**		
Urban	36.6	1,633
Rural	63.4	2,833
**Educational level**		
No education	10.4	466
Primary	51.3	2,292
Secondary	33.8	1,510
Higher	4.4	197
**Wealth status**		
Poor	45.4	2,027
Middle	19.1	853
Rich	35.5	1,586
**Employment statu**s		
Unemployed	49.7	2,221
Employed	50.3	2,245
**Age at first marriage**		
< 18	46.2	2,064
18 +	53.8	2,401
**Ever used any contraceptive method**		
No	18.9	842
Yes	81.1	3,623
**Children ever born**		
1–2	36.6	1,635
3–4	29.9	1,337
5 +	33.4	1,493
**Ideal number of children**		
0	0.9	38
1–2	5.5	241
3–4	40.3	1,765
5 +	53.3	2,333
**Reproductive health decision-making capacity**		
No	49.7	2,217
Yes	50.3	2,248
**Knows fertile period**		
No	80.2	3,582
Yes	19.8	884
**Ever experienced any sexual violence**		
No	85.1	3,802
Yes	14.9	663
**Unintended pregnancy**		
Intended	35.5	2.880
Unintended	64.5	1,586
Total	100	4,465

### Characteristics of women experiencing unintended pregnancy

Table 2 shows a summary of results of chi-squared analysis comparing the variation of socio-economic and demographic characteristics of women with unintended pregnancy. The following variables were found to be associated with unintended pregnancy (*p* < 0.05): age, education level, wealth status, employment status, ever use of contraception, children ever born, reproductive health decision making capacity and ever experience of sexual violence.

**Table 2:** Socio-economic and demographic characteristics of women experiencing unintended pregnancies

**Characteristics**	**Unintended pregnancies**		
	%	95% CI	*P*-value
**Age group**			0.000
15–19	43.1	[36.1,50.4]	
20–24	37.4	[34.0,41.0]	
25–29	31.5	[27.9,35.3]	
30–34	29.6	[26.4,33.1]	
35–39	39.4	[34.4,44.7]	
40–44	40.2	[33.4,47.3]	
45–49	49.3	[38.0,60.6]	
**Type of residence**			0.507
Urban	37.4	[33.5,41.5]	
Rural	34.4	[32.0,36.9]	
**Educational level**			0.002
No education	36.6	[31.7,41.7]	
Primary	36.5	[34.0,39.0]	
Secondary	36.2	[32.0,40.6]	
Higher	16.6	[10.7,25.0]	
**Wealth status**			0.027
Poor	34.7	[32.2,37.3]	
Middle	34.4	[30.4,38.7]	
Rich	37.1	[32.7,41.8]	
**Employment status**			0.005
No	38.1	[35.5,40.8]	
Yes	32.9	[30.0,35.9]	
**Age at first marriage**			0.748
< 18	35.8	[33.2,38.6]	
18 +	35.2	[32.4,38.2]	
**Ever used any contraceptive method**			0.000
No	28.6	[25.0,32.5]	
Yes	37.1	[34.7,39.6]	
**Children ever born**			0.000
1–2	34.3	[31.0,37.7]	
3–4	29.5	[26.5,32.6]	
5 +	42.3	[39.2,45.4]	
**Ideal number of children**			0.334
0	39.6	[23.3,58.5]	
1–2	41.6	[33.8,49.8]	
3–4	36.2	[32.5,40.0]	
5 +	34.4	[32.0,36.9]	
**Reproductive health decision-making capacity**			0.000
No	36.7	[33.5,39.9]	
Yes	34.4	[31.9,36.9]	
**Knows fertile period**			0.110
No	36.3	[33.9,38.7]	
Yes	32.5	[28.4,36.7]	
**Ever experienced any sexual violence**			0.000
No	33.5	[31.2,35.8]	
Yes	47.2	[42.4,52.0]	
Total	35.5	[33.4,37.7]	*n* = 4,465

Unintended pregnancies were significantly higher among women 45 to 49 years (49%), those that had no education (37%) and those with primary level of education (37%). More women from a rich wealth quintile index (37%) reported to have experienced unintended pregnancies compared to other wealth quintile categories. Similarly, unintended pregnancies were higher among women who were unemployed (38%), those that ever used any contraceptive method (37%), those that had five or more children (42%), those that had no reproductive health decision-making capacity (38%) and those that experienced sexual violence (47%).

### Factors associated with unintended pregnancy

The results of Model 1, (which is the unadjusted odds ratios (UORs)) show that, women who had experienced sexual violence were 1.77 times [CI 1.42—2.22] more likely to have unintended pregnancies. Model II shows the adjusted odds ratios (AORs) of unintended pregnancy controlled for use of contraception and reproductive health decision-making capacity of women. Results show that women who had ever used contraceptive methods were 1.50 times [CI 1.20—1.87] more likely to have an unintended pregnancy adjusted for reproductive health decision-making capacity. Reproductive health decision-making capacity of women is not associated with unintended pregnancies (Table 3).

**Table 3:** Logistic regression results on sexual violence, contraception and reproductive health decision-making capacity and unintended pregnancies

Characteristics	Model I		Model II		Model III	
	**OR**	**CI**	**AOR**	**CI**	**AOR**	**CI**
**Ever experienced any sexual violence**						
No (RC)	1					
Yes	1.77***	1.42—2.22			1.73***	1.38—2.17
**Ever used any contraceptive method**						
No (RC)			1		1	
Yes			1.50***	1.20—1.87	1.47***	1.17—1.84
**Reproductive health decision-making capacity**						
No (RC)			1		1	
Yes			0.88	0.74—1.04	0.90	0.76—1.08
Constant	0.50***	0.45—0.56	0.42***	0.35—0.51	0.39***	0.32—0.47

^***^ *p* < 0.001, ** *p* < 0.01, * *p* < 0.05

Model III shows the AORs of unintended pregnancies controlled for sexual violence, ever use of contraception and reproductive health decision-making capacity of women. When the Model III results are compared to the Model II results, there is a minor decrease in the risk of unintended pregnancy among women who had experienced sexual violence. However, women who had experienced sexual violence were (still) more likely to have an unintended pregnancy than those who had not [AOR: 1.73, CI 1.38—2.17]. On the other hand, there was no significant statistical association between unintended pregnancies and reproductive health decision-making capacity of women.

Further, our study performed some mediation analysis to help us understand how sexual violence through the exposure variables namely ever use of contraception and women’s RHDMC influence the outcome variable—unintended pregnancy. The first mediation model (Table 4) shows that sexual violence was significantly associated with ever use of contraception (path a_1_, ß = 0.357, *p* = 0.001) and unintended pregnancy (path c, ß = 0.594, *p* < 0.001); and ever use of any contraception was equally associated with unintended pregnancy (path b_1_, ß = 0.210, *p* = 0.006).

**Table 4:** Associations between exposure, mediators and outcome variable

**Mediation Models**	ß	*P*-value
**Mediation Model I**		
Ever used any contraception and sexual violence (path a_1_’)	0.357	0.001
Unintended pregnancy and sexual violence (path b_1_’)	0.594	0.000
Unintended pregnancy and ever used any contraception controlled for sexual violence (path c')	0.210	0.006
**Mediation Model II**		
RHDMC and sexual violence (path a_2_’)	-0.323	0.000
Unintended pregnancy and sexual violence (path b_1_’)	0.603	0.000
Unintended pregnancy and RHDMC controlled for sexual violence (path c')	-0.005	0.939
**Mediation Model III**		
Ever used any contraception and sexual violence (path a_1_’)	0.391	0.000
Ever used any contraception and RHDMC (path d’)	0.403	0.000
Unintended pregnancy and Sexual violence and Sexual violence (path c’)	0.592	0.000
Unintended pregnancy and Ever use of any contraception (path b_1_’)	0.211	0.005
Unintended pregnancy and RHDMC (path b_2_’)	-0.018	0.767
RHDMC and sexual violence (path a_2_’)	-0.323	0.000

In the second mediation model shown in Table 4, the coefficient for sexual violence in relation to unintended pregnancy slightly increased in magnitude and significance (path c, ß = 0.603, *p* < 0.001). Equally, sexual violence was significantly negatively associated with women’s RHDMC (path a_1_, ß = -0.323, *p* < 0.001) while the association between women’s RHDMC and unintended pregnancies was not significant (path b_1_, ß = -0.005, *p *= 0.939).

The third model combined the main independent variable sexual violence; and the two mediating variables and how they all interacted in predicting unintended pregnancy. After controlling for both ever used any contraception and women’s RHDMC, sexual violence is still significantly associated with unintended pregnancy (path c, ß = 0.592, *p* < 0.001). In addition, controlling for women’s RHDMC, results indicate that there was a positive significant association between sexual violence and unintended pregnancy and ever used any contraception and unintended pregnancy (path a_1_, ß = 0.391, *p* < 0.001 and path b_1_, ß = 0.211, *p* = 0.005). On the contrary, a negative significant association was observed between sexual violence and women’s RHDMC (path b_2_, ß = -0.323, *p* < 0.001).

Model IV shows the AORs of unintended pregnancies controlled for all covariates. The magnitude of the effect between sexual violence and unintended pregnancies decreased slightly. Despite the decrease in association, results show that women who had ever experienced sexual violence were 1.74 times [CI 1.38—2.19] more likely to have had an unintended pregnancy. Results by age group of women show that all women 20–49 years were less likely to have had an unintended pregnancy when compared with younger women 15–19 years. Women that had attained higher education [AOR 0.38; CI 0.21—0.69] and those who were employed [AOR 0.81; CI 0.69—0.94] were less likely to have experienced an unintended pregnancy (Table 5).

**Table 5:** Logistic regression results on sexual violence, selected socio-economic and demographic factors and unintended pregnancies

Characteristics	Model IV	
	**AOR**	**CI**
**Ever experienced any sexual violence**		
No (RC)		
Yes	1.74***	1.38—2.19
**Age group**		
15–19 (RC)		
20–24	0.64*	0.44—0.94
25–29	0.39***	0.26—0.57
30–34	0.28***	0.19—0.43
35–39	0.35***	0.22—0.55
40–44	0.35***	0.21—0.57
45–49	0.50*	0.25—0.98
**Type of residence**		
Urban (RC)		
Rural	0.89	0.68—1.16
**Educational level**		
No education (RC)		
Primary	0.95	0.77—1.17
Secondary	0.94	0.70—1.26
Higher	0.38**	0.21—0.69
**Wealth status**		
Poor (RC)		
Middle	0.98	0.79—1.22
Rich	1.28	0.95—1.73
**Employment statu**s		
Unemployed (RC)		
Employed	0.81**	0.69—0.94
**Age at first marriage**		
< 18 (RC)		
18 +	1.25**	1.06—1.48
**Ever used any contraceptive method**		
No (RC)		
Yes	1.48**	1.16—1.88
**Children ever born**		
1–2 (RC)		
3–4	1.28	1.00—1.63
5 +	2.83***	1.96—4.07
**Ideal number of children**		
0 (RC)		
1–2	1.32	0.55—3.18
3–4	1.01	0.46—2.20
5 +	0.79	0.36—1.71
**Reproductive health decision-making capacity**		
No (RC)		
Yes	0.90	0.76—1.08
**Knows of fertile period**		
No (RC)		
Yes	0.94	0.77—1.15

^***^ *p* < 0.001, ** *p* < 0.01, * *p* < 0.05

On the other hand, women who first married at 18 years and above [AOR 1.25; CI 1.06—1.48], ever used any contraceptive method [AOR 1.48; CI 1.16—1.88] and had five or more children [AOR 2.83; CI 1.96—4.07] were more likely to have experienced an unintended pregnancy. However, place of residence, ideal number of children, reproductive health decision making capacity and correct knowledge of fertility period were not significantly associated with unintended pregnancies.

## Discussion

According to the current paper’s findings, married women in Zambian had a high rate of unintended pregnancies (36%).This figure is much higher than what was found in a study of the 2016 Ethiopia DHS, where 26% of women had unintended pregnancies [25]. The prevalence of unintended pregnancies in Zambia, on the other hand, is lower than that of Uganda, where 38% of the women in a study of the 2016 DHS had an unintended pregnancy [26].

Using data from the 2018 Zambia DHS, we investigated the association between sexual violence and unintended pregnancy. Forty-seven percent of women who had ever experienced spousal sexual violence had unintended pregnancy. Results of both the UORs and AORs show a significant association between experience of any sexual violence and unintended pregnancies among married women in Zambia. Unintended pregnancy was 1.7 times more likely to happen in women who had experienced any form of sexual violence from a spouse than in women who had not experienced any. Our findings are similar to other studies where women who had experienced sexual violence had a 1.6, 1.7 and 2.3 times higher likelihood of unwanted pregnancies than women who had never experienced sexual violence [24, 27, 28]. There are a number of possible explanations for why this situation happens. Failure to meet a husband's sexual demands, for example, might lead to arguments and forced and unprotected sex, resulting in pregnancies from such an experience being reported to be unwanted [28]. Furthermore, IPV fosters an environment that influences a woman's participation in decision-making related to her own health care, availability and use of contraceptives, and bargaining for safe sex, such as condom use, leading to forced unprotected sex and consequently unwanted pregnancy [12, 13]. Others claim that women are usually subjected to sexual exploitation and torture, which has long-term harmful consequences for their mental, physical, reproductive, and sexual health [5].

Our study found that unintended pregnancies were less common among married women age 20 to 49 than among those age 15 to 19. This is backed with the generally held view that young married women have a higher risk of experiencing an unintended pregnancy due to a number of inadequacies, which include inability to negotiate safe sex. It may also be due to the fact that the majority of young married women may have little or no awareness of sexuality and family formation practices, which are only learnt after they have been married [28, 29]. Moreover, for such young married women, husbands tend to take it as though sexual intercourse is their entitlement, as such, they have all the right to do whatever they want with regard to sexual life even to their own wives, leading to use of force and other forms of sexual violence thereby increasing the likelihood of unintended pregnancies [30, 31].

Furthermore, the current social cultural norms and beliefs within society such as early marriages and traditional teachings including those which prepare young women for marriage have perpetuated this practice, thus the higher likelihood of unintended pregnancies among young women age 15–19 years [30]. Other studies, on the other hand, reveal that older women between the ages of 40 and 44 years, as well as those between the ages of 45 and 49 years, are more likely than younger women to have unintended pregnancies [24, 29]. This is because women in these age groups may have had the desired number of children, thus, any pregnancy experienced would be unwanted. Women above the age of 35 years have a higher risk of maternal death, baby death, and induced abortions. Furthermore, in resource-poor nations like Zambia, such women may be at risk of reproductive health practices and behaviour, including low contraceptive usage, low prenatal attendance, and non-facility births [32].

Studies have shown that women who have attained high school or tertiary education are less likely to experience sexual violence and, as a result, unintended pregnancy is also less likely. This finding is comparable to what our study established, where women with higher education had lower risk of having unintended pregnancies. Various reasons could be advanced for this observed phenomenon, to the effect that women with higher education have better understanding of their rights and thus are able to bargain their way out or speak with their spouse when the risk of sexual violence is eminent. Furthermore, unlike uneducated married women, educated married women may be able to access family planning services, use contraceptives correctly and consistently and thereby reduce odds of unintended pregnancy [33].

Our findings further show that women who married when they were 18 years old or older had a higher chance of having an unintended pregnancy than women who married when they were younger than 18 years old. This finding contradicts a 2015 study in India, which showed that unplanned pregnancies decreased with increasing age at marriage among currently pregnant ever-married women [33]. It is also at variance with a study in Damot Gale District, Southern Ethiopia where women who married later in life were less likely to have an unintended pregnancy [14]. Holding all else constant, it is assumed that women who marry later in life are more likely to have attained some secondary or higher education and are likely to be income earners and may therefore have control of their reproductive lives and would protect themselves against unintended pregnancies. The possible explanation for this finding in our study is that whilst it is expected that women who marry later, maybe knowledgeable enough on how they can prevent unintended pregnancies compared to those who get married at a young age, there is a variance in having knowledge on contraception methods and actual practice as behaviour takes time to adjust especially in a country like Zambia with a deep rooted cultural and societal beliefs that married couples should bear children.

Our results were somehow surprising in regards to those women who reported ever use of any kind of contraception; they had 1.48 times the chance of having an unintended pregnancy compared with women who had never used any form of contraception. This finding, although surprising to say the least, is consistent with study findings in India and Ivory Coast [33, 34]. Moreover, most married women in Zambia are using contraception for spacing the births rather than to limit the number of children. Furthermore, injectables and pills are the most used methods of contraception among married women [19]. These short-term contraceptive methods, like others if not correctly and/or consistently used may result in failure, increasing the likelihood of women having mistimed births despite ever use of any contraception. In addition, societal and cultural beliefs and norms about contraceptive methods among married women, and so on, could explain this finding. Contraceptive discontinuation could also be a contributing factor to unintended pregnancy [14, 34].

The odds of unintended pregnancy was higher among women with high parity (5 and more children ever born). Similarly, another study found that the odds of unintended pregnancy was significantly higher among women with more than two children ever born [13]. The likelihood of this occurring in a country like Zambia is highly possible since 20% of currently married women have an unmet need for family planning. Furthermore, certain women may be looking forward to having a child of a specific sex, and once this desire is fulfilled, the need for children would be drastically reduced. Moreover, because some males prefer a specific sex of a child, usually "males," the odds of women having unwanted pregnancies may persist until their partner's wish is met.

Results in this study have shown that factors such as place of residence and wealth status were not significantly associated with unintended pregnancies. This finding contradicts a study in Malawi where it was found that fertility preference and the number of children ever born have an influence on mistimed pregnancies and also that women's age, wealth status, fertility preference, and residence all increased the likelihood of an unwanted pregnancy [35].

Our study found no significant association between unintended pregnancy and reproductive health decision-making capacity. In addition, the prevalence of unintended pregnancy was 37% among women who had no reproductive health decision-making capacity and 34% among women who had reproductive health decision-making capacity. This finding is different from other studies where women who had the capacity to make reproductive health decisions were less likely to have experienced unintended pregnancies compared to those who did not have the capacity [21]. Therefore, further research is required to explore why such a contradictory finding in Zambia.

### Limitations of the study

The cross-sectional study design of the ZDHS prohibits us from undertaking a causal study between the dependent and independent variables, which would have been more appropriate. Furthermore, due to the nature of the ZDHS data, it was not possible to obtain qualitative data on the social and cultural factors associated with sexual violence and unintended pregnancies. Sexual violence is a sensitive issue and is subject to misreporting. The DHS asks standard questions and follows the World Health Organization’s guidelines for collecting information on domestic violence in an ethical manner. As a result, we are confident that data collected gives reliable estimates of women in Zambia who have been victims of sexual violence. Lastly, the retrospective classification of births in the last five years prior to the survey as wanted or unwanted may be subject to change overtime and be subject to recall bias. A woman whose pregnancy was unintended (mistimed or not wanted any more) may change the status to wanted pregnancy after giving birth.

## Conclusion

Our study has established that, in comparison to other nations, Zambia has a high prevalence of unintended pregnancies, particularly among women who have experienced sexual violence. Women in Zambia who have experienced sexual violence are about twice more likely than those who have not to have unintended pregnancies. Other predictors of unintended pregnancy were first marriage at age 18 or older, ever-used contraceptives, and with high parity (5 +) were associated with unintended pregnancies. On the other hand, women who were age 20–49, had attained higher education and were in employment were protective of unintended pregnancy.

Measures aiming at eliminating gender disparity and early detection of sexual violence should be prioritized and integrated into the existing family planning services provided by the Ministry of Health's Maternal and Child Health Department, rather than being offered as a vertical service. If Zambia is to attain Sustainable Development Goal (SDG) 3—Good health and well-being—and SDG 5—Gender Equality, health service provision must take into account requirements of various categories of women, such as adolescents and the less educated. Furthermore, publicizing the importance of women reporting acts of sexual violence to relevant authorities should be prioritized to reduce not only unwanted births but also prevent future recurrence of sexual violence among married women.

## Data Availability

The dataset supporting the conclusions of this article can be accessed following clearance after written request at https://dhsprogram.com/Data/. Dataset analysed during the current study are available from the corresponding author on reasonable request (mwewa.kas@gmail.com).

## Abbreviations

AORs: Adjusted Odds Ratios
CI: Confidence Interval
DHS: Demographic and Health Survey
EA: Enumeration Area
IPV: Intimate Partner Violence
IRBs: Institutional Review Boards
OR: Odds Ratio
RC: Reference Category
STIs: Sexually Transmitted Infections
SDG: Sustainable Development Goal
TDRC: Tropical Diseases Research Centre
UIs: Uncertainty Intervals
WHO: World Health Organisation
ZDHS: Zambia Demographic and Health Survey
